# Growth Control of *Listeria monocytogenes* in Raw Sausage via Bacteriocin-Producing *Leuconostoc carnosum* DH25

**DOI:** 10.3390/foods13020298

**Published:** 2024-01-17

**Authors:** Andrea Tönz, Susette Freimüller Leischtfeld, Marc J. A. Stevens, Deborah Glinski-Häfeli, Valentin Ladner, Corinne Gantenbein-Demarchi, Susanne Miescher Schwenninger

**Affiliations:** 1 ZHAW Zurich University of Applied Sciences, Institute of Food and Beverage Innovation, Food Biotechnology Research Group, 8820 Wädenswil, Switzerland; andrea.toenz@zhaw.ch (A.T.); susette.freimueller@zhaw.ch (S.F.L.); deborah@glinski.ch (D.G.-H.); valentin.ladner@gmail.com (V.L.); c.gantenbein.demarchi@gmail.com (C.G.-D.); 2 University of Zurich, Vetsuisse Faculty, Institute for Food Safety and Hygiene, 8057 Zurich, Switzerland; mstevens@fsafety.uzh.ch

**Keywords:** bacteriocin, leucocin, *Leuconostoc carnosum*, *Listeria monocytogenes*, biocontrol, raw sausage

## Abstract

The current study addresses the critical issue of *Listeria monocytogenes* growth in raw sausage/meat products leading to human infections, most commonly listeriosis, which is known for its high fatality rate. This research focuses on the isolation, identification, and screening of lactic acid bacteria from various meat and fish products in Switzerland. In total, 274 lactic acid bacteria strains were isolated from 30 different products and were screened for their ability to inhibit *Listeria monocytogenes* growth, with 51 isolates demonstrating anti-*Listeria* activity at 8 °C, 15 °C, 25 °C, and 37 °C. Further experiments, using a meat model and a raw sausage challenge test, demonstrated that *Leuconostoc carnosum* DH25 significantly inhibited *Listeria monocytogenes* growth during the ripening and storage of the tested meat/sausage. This inhibitory effect was found to be attributed to the bacteriocins produced by *Leuconostoc carnosum* DH25 rather than factors like pH or water activity. The stability of the anti-*Listeria* substances was examined, revealing their resistance to temperature and pH changes, making *Leuconostoc carnosum* DH25 a promising protective culture for raw sausages. The genome sequencing of this strain confirms its safety, with no antibiotic resistance genes or virulence factors detected, and reveals the presence of the structural genes for the production of the bacteriocin LeucocinB-Ta11a. This study underscores the potential of LAB strains and their bacteriocins as effective tools for enhancing food safety and preventing *Listeria monocytogenes* growth in meat products, offering valuable insights into biocontrol strategies in the food industry.

## 1. Introduction

The control of *Listeria monocytogenes* (*L. monocytogenes)* growth in food is essential to prevent infection in humans, which can cause listeriosis, a severe infection with a particularly high case-fatality rate [1]. In 2020, 1876 invasive human cases of *L. monocytogenes* were confirmed, which caused 780 hospitalizations and 167 deaths in the EU [2]. For healthy individuals, an infection may lead to mild gastroenteritis, but for immunocompromised individuals, it can result in serious invasive diseases [3]. *L. monocytogenes* is a ubiquitous, foodborne pathogen and is considered a high-risk organism affecting the safety of various foods, including raw meat and fish products [4]. Fermented raw sausages show several barriers against undesirable pathogens and spoilage microorganisms, such as a low pH, high salt levels, the presence of organic acids, and low water activity. However, *L. monocytogenes* is resistant to those barriers and is able to grow in raw sausages [5]. There are several strategies that can be used to control the growth of *L. monocytogenes* during the ripening of raw sausage. One of these strategies is biocontrol, where microorganisms and their metabolites are used to extend the shelf life and improve the safety of a food product [6]. The naturally present lactic acid bacteria (LAB) are the basis for the fermentation process of raw sausage. Besides their ability to reduce the pH of the product during fermentation and consequently retard the growth of spoilage and pathogenic microorganisms, LAB are also able to produce antimicrobial compounds, including pediocin-like bacteriocins, with a high specific activity against *L. monocytogenes.* As a result, LAB are promising biocontrol agents aimed at increased food safety concomitant with reduced use of artificial additives. An important prerequisite for the antimicrobial effect of protective cultures in fermented products is their ability to grow as well as produce bacteriocins in these products [7,8]. Nisin and pediocin PA-1 (also known as Pediocin AcH) are the most studied bacteriocins with broad activity against Gram-positive bacteria, particularly *L. monocytogenes*. Both bacteriocins are widely used in the form of protective cultures, whereas nisin is also approved in many countries as a food additive [9]. However, nisin and pediocin AcH/PA-resistant *L. monocytogenes* strains have been detected, and cross-resistances are possible [10,11,12]. Therefore, it is essential for biocontrol concepts and food safety to screen for LAB with anti-*Listeria* activity that is based not only on the production of nisin or pediocin AcHPA-1 but also on the production of different bacteriocins, including novel bacteriocins. The aim of this study was to develop a protective culture active against human pathogenic serovars of *L. monocytogenes* (1/2a, 1/2b, 4b) for use in raw sausage production. To avoid bacteriocin resistance and to broaden the spectrum of activity, the inhibition ability of the protective culture should be based on more than one bacteriocin or, even better, on a novel bacteriocin, and the inhibition of *L. monocytogenes* should also be consistent at low temperatures during the storage of raw sausage.

## 2. Materials and Methods

### 2.1. Isolation of LAB Strains

LAB strains were isolated from thirty meat and fish products (Table 1), produced without starter or protective cultures. For the enumeration of LAB, an aliquot of each meat or fish product was weighed into a stomacher bag containing a filter and homogenized in a 1:10 ratio of the diluent (0.1% peptone bacteriological, 0.85% NaCl). Decimal dilutions of the homogenate were prepared in the diluent and 0.1 mL of the appropriate dilutions were surface-plated on an Elliker agar (Difco^TM^ Elliker Broth, Becton Dickinson, Allschwil Switzerland; 1.5% Agar Bios Special LL, Biolife Italiana, Monza, Italy); MRS agar (Carl Roth, Karlsruhe, Germany); D-MRS agar (1% peptone bacteriological, Biolife Italiana, Monza, Italy; 0.5% yeast extract, Merck, Buchs, Switzerland; 0.5% meat extract, Merck, Buchs, Switzerland; 2% D(+)-sucrose, Carl Roth, Karlsruhe, Germany; 0.1% tween 80,Merck, Buchs, Switzerland; 0.2% K_2_HPO_4_, Merck, Buchs, Switzerland; 0.2% triammonium citrate, Merck, Buchs, Switzerland; 0.1 g/L MgSO_4_·7H_2_O Carl Roth, Karlsruhe, Germany, 0.038 g/L MnSO_4_·H_2_O, Merck, Karlsruhe, Germany and 1.5% Agar Bios Special LL, Biolife Italiana, Monza, Italy) with the pH of D-MRS agar adjusted to 8.5 before sterilization using 1 M of NaOH (Fluka Honeywell, Morris Plains, NJ, USA) [13]. The plates were incubated at 30 °C for 3 days under anaerobic conditions and subsequently enumerated. Up to 20 colonies per product of presumptive LAB isolates were selected randomly and then purified by three successive streaking steps on agar plates of the same medium. The purified LAB isolates were integrated into the culture collection of the Food Biotechnology Research Group of the ZHAW Zurich University of Applied Sciences and stored at −80 °C in MRS broth containing 25% (*v*/*v*) glycerol.

### 2.2. Identification of Presumptive LAB Isolates

The identification of LAB isolates was performed using MALDI-TOF MS as described by Miescher Schwenninger et al. (2016) [14]. The extraction of MALDI-TOF MS was conducted by picking single colonies of each isolate and transferring each to 100 µL of sterile double-distilled water. This was followed by 300 µL of ethanol addition and centrifugation (13,000× *g*, 20 °C, 2 min); the supernatant was then removed, and 50 µL of 70% acetic acid was added, and the suspension was mixed. The same amount of acetonitrile was added, mixed, and centrifuged as described above. The obtained supernatant was then analyzed using MALDI-TOF MS, Version 3.1 Biotyper (Bruker Daltonics, Bremen, Germany) with 6903 entries.

### 2.3. Screening for Antimicrobial Activity

#### 2.3.1. Antimicrobial Screening by Agar Spot Assay

A modified version of the agar spot assay described by Inglin et al. (2015) [15] was used to assess the anti-*Listeria* activity of isolated presumptive LAB strains and to assess whether they exhibit any negative activity against commercially available starter cultures (Moguntia Food Group, Gossau, Switzerland) representing common species of meat starter cultures. A meat simulation medium (MSM+) was modified from Leroy & de Vuyst (2005) [16], where the sodium chloride and nitrite concentrations were adapted and the media was composed of 9 g/L of the meat extract (Carl Roth, Karlsruhe, Germany), 13 g/L of D(+)-glucose (Carl Roth, Karlsruhe, Germany), 2 g/L of yeast extract (Carl Roth, Karlsruhe, Germany), 0.04 g/L of manganese(II)-sulfate monohydrate (Merck, Buchs, Switzerland), 5 g/L of lactic acid (Carl Roth, Karlsruhe, Germany), 30 g/L of sodium chloride (Carl Roth, Karlsruhe, Germany), 0.15 g/L of sodium nitrite (Carl Roth, Karlsruhe, Germany), 11 g/L of peptone bacteriological (Biolife Italiana, Monza, Italy), and 1 g/L of Tween 80 (Merck, Buchs, Switzerland) solidified with 15 g/L agar (Biolife Italiana, Monza, Italy). The media were filled in each well of a 6-well plate (TPP AG, Trasadingen, Switzerland). Each well was inoculated by single LAB strains using a sterile toothpick to transfer colonies, and the plates were incubated anaerobically at 30 °C for 72 h. Then, each well was overlaid with 0.9 mL of brain–heart infusion (BHI) (Biolife Italiana, Monza, Italy) soft agar containing 0.6% (*w*/*v*) agar that was inoculated with 7 log CFU/mL of *L. monocytogenes* or a meat starter strain. The 6-well plates were incubated aerobically at 8 °C, 15 °C, 25 °C, and 37 °C for 1, 2, 4, and 7 days and the inhibition of *L. monocytogenes* was evaluated by measuring the zone of inhibition starting from the edge of the colony to the edge of the halo, applying the following classification: 0–0.9 mm of halo (−), 1–2.9 mm of halo (+), 3–4.9 mm of halo (++), 5–6.9 mm of halo (+++), 7–14.9 mm of halo (++++), and >14.9 mm of halo (++++S). All presumptive LAB strains were screened against *L. monocytogenes* strains ATTC 15313 (serovar 1/2a), SLCC 27555 (serovar 1/2b), and ATCC 19115 (serovar 4b), and against meat starter culture strains *Staphylococcus carnosus* 50006, *Pediococcus pentosaceus* 50005, *Latilactobacillus sakei* 50003, *Leuconostoc citreum* 50018, *Latilactobacillus sakei* 50019, *Staphylococcus carnosus* 50020, *Staphylococcus xylosus* 50001, *Staphylococcus xylosus* 50002, and *Debaromyces hansenii* 50007, obtained from Moguntia Food Group (Gossau, Switzerland). Screening was performed in duplicates.

#### 2.3.2. Anti-*Listeria* Screening by Well Diffusion Assay

Presumptive LAB isolates showing anti-*Listeria* activity in the agar spot assay were further characterized by a modified well diffusion assay (WDA), according to Tagg and McGiven (1971) [17]. LAB strains were incubated for 48 h at 30 °C in MSM+ broth, and cell-free supernatants (CFS) were prepared via the centrifugation of the cultures for 10 min at 8000× *g*, 4 °C. This was followed by the sterile filtration of the supernatant through a 0.22 µm filter. The pH of the CFS was set to 7 with 10M NaOH, and the CFS was stored at 4 °C for a maximum of 5 days. For the WDA, 25 mL of BHI soft agar containing 0.8% (*w*/*v*) agar, inoculated with 6 log CFU/mL of *L. monocytogenes,* was filled into a sterile Petri dish. After solidification, four 6.5 mm diameter wells per plate were cut out using a cork borer. The wells were filled with 100 µL of the CFS and incubated for 24 h at 37 °C. Inhibition was shown by a clear zone around the 6.5 mm wells and was measured from the edge of the well to the edge of the inhibition zone, designated as the radius and shown in mm. Uninoculated MSM+ broth was used as a negative control. The well diffusion assay was performed with three independent CFSs of each LAB strain, and LAB strains were screened against the *L. monocytogenes* strains ATTC 15313 (serovar 1/2a), SLCC 27555 (serovar 1/2b), and ATCC 19115 (serovar 4b).

### 2.4. Characterization of Anti-Listeria Substances Produced by Selected LAB

#### 2.4.1. Sensitivity of Anti-*Listeria* Metabolites to Proteolytic Enzymes

Anti-*Listeria* active metabolites of the LAB isolates were examined for their sensitivity to proteinase K (AppliChem GmbH, Darmstadt, Germany), papain (Carl Roth, Karlsruhe, Germany), and trypsine (Carl Roth, Karlsruhe, Germany), using a well diffusion assay, as described above. Three 6.5 mm wells were cut in line, ensuring a 6 to 8 mm distance between each. In total, 100 µL of a proteolytic enzyme solution, which contained 100 µL of the corresponding enzyme (1 mg/mL of a solution of 5 µL of calcium chloride solution 1 M and 895 µL of dH_2_O), was placed into the middle well, and the outer wells were filled with 100 µL of CFS. Plates were incubated for 24 h at 37 °C. The enzyme degradation of anti-*Listeria* active metabolites in the CFS was shown by a characteristic crescent-shaped zone of inhibition, opened toward the proteolytic enzyme. Uninoculated MSM+ broth was used as a negative control. The well diffusion assay was performed with three independent CFSs of each LAB.

#### 2.4.2. Determination of the Presence of Structural Genes for Bacteriocins

In total, 51 of the isolated LAB strains showing anti-*Listeria* activity in the agar spot assay and WDA were screened for the presence of structural genes of known bacteriocin using a polymerase chain reaction (PCR). All primers and annealing temperatures are listed in Supplementary Table S1; primer pairs were purchased from Microsynth AG (Balgach, Switzerland).

The PCR reaction mix for each sample was prepared as follows: 1 µL of the template (1 colony of the appropriate strain in 100 µL of sterile double-distilled H_2_O), 1 µL of each 10 µM forward/reverse primer, 10 µL of the KAPA Taq ReadyMix PCR kit (KAPA BIOSYSTEMS, Wilmington, MA, USA), and 7 µL of sterile double-distilled H_2_O. The amplification was performed in the thermocycler SENSOQUEST Labcycler (SensoQuest GmbH, Land, Göttingen, Germany) with the following program: initial denaturation: 95 °C for 5 min; amplification (35 cycles); denaturation: 95 °C for 30 s; annealing temperature (AT) specific for each primer pair (Supplementary Table S1); extension: 72 °C for 10 min; final extension: 72 °C for 10 min. The PCR products were then run in a 1% agarose gel electrophoresis.

### 2.5. Challenge Test in a Meat Model and in Raw Sausage

The anti-*Listeria* activity of 39 LAB strains belonging to the species *Latilactobacillus sakei* (n = 30), *Lactiplantibacillus plantarum* (n = 6), *Leuconostoc carnosum* (n = 1), and *Latilactobacillus curvatus* (n = 2) was determined in a meat model challenge test in Petri dishes, consisting of 20 g of minced meat, 2.8 g of nitrite curing salt (Roth GmbH), 0.06 g of a starter culture BessaStart (Moguntia Food Group, Gossau, Switzerland), and 1 g of salami spice (Moguntia Food Group, Gossau, Switzerland) per Petri dish, which was incubated for four days at a temperature profile simulating the 14 days ripening process of raw sausage in fast motion (4.5 h at 22 °C, 21 h at 24 °C, 14 h at 22 °C, 14 h at 20 °C, 42 h at 18 °C). Furthermore, *Leuconostoc carnosum* DH25 (*Lc. carnosum* DH25) and *Latilactobacillus sakei* DH42 (*Lcb. sakei* DH42) DH42 were applied in a challenge test in two different raw sausages, Salami and Mettwurst, containing minced meat (Salami: 96.52% (*w*/*w*)/Mettwurst: 97.29% (*w*/*w*)), nitrite curing salt (Salami: 2.8% (*w*/*w*)/Mettwurst: 2.4% (*w*/*w*)), dextrose (Salami: 0.65% (*w*/*w*)/Mettwurst: 0.25% (*w*/*w*)) and 0.03% (*w*/*w*) of the starter culture PrestoStart (Moguntia Food Group) was filled in Fibrous casings with a caliber of 40, and ripened for 14 days with the temperature-relative humidity–time profile shown in Table 2. Both types of samples, the meat model in Petri dishes and the raw sausages, were inoculated with 5.0 log of CFU/g *L. monocytogenes* N18-440 (Institute for Food Safety and Hygiene, Vetsuisse Faculty, University of Zurich, Zurich, Switzerland) and 6.3 log of CFU/g LAB. To evaluate the inhibition capacity at lower temperatures, *Lc. carnosum* DH25 was applied in a meat model challenge test at 8 °C and 12 °C for 14 days of storage.

During the simulation of the 14-day ripening process in fast motion, samples of the meat model challenge test were taken for the analysis of cell counts on days 0, 2, and 4. The samples of the raw sausage challenge were tested on days 0, 8, 15, and 36 for salami and on days 0, 1, 15, and 29 for Mettwurst, respectively. Samples from the meat model challenge test performed at 8 °C and 12 °C were analyzed on days 0, 4, 8, and 15. All samples were mixed with a diluent (1 g/L peptone and 8.5 g/L NaCl) in a ratio of 1:10, and 0.1 mL of decimal dilutions were plated on MRS agar for cell counts of LAB and on Agar Listeria according to Ottaviani and Agosti (ALOA) (Biolife Italiana, Monza, Italy) for *L. monocytogenes*, respectively. MRS agar plates were incubated anaerobically at 30 °C for 72 h and ALOA plates aerobically at 37 °C for 48 h.

Physicochemical characteristics during storage were analyzed as follows: Samples of the meat model challenge test were analyzed for water activity (LabMaster-a_w_, Novasina, Lachen, Switzerland) and pH (pH-Meter 761 Calimatic, Knick, Zofingen, Switzerland) on days 0, 2, and 4. Samples from the raw sausage challenge test were analyzed on days 0, 8, 15, and 36 for salami and on days 0, 1, 15, and 29 for Mettwurst, respectively. Samples from the meat model challenge test at 8 °C and 12 °C were analyzed on days 0, 4, 8, and 15. The water activity of only the uninoculated, *Listeria*-free samples was measured, due to safety reasons.

### 2.6. Characterization of the Bacteriocin Produced by Leuconostoc carnosum DH25

#### 2.6.1. Stability and Activity of the Anti-*Listeria* Substance of *Leuconostoc carnosum* DH25 in Various Conditions

The activity of the anti-*Listeria* substance of *Lc. carnosum* DH25 was investigated in MSM+ medium after incubation at 4 °C/21 days, 8 °C/10 days, 12 °C/10 days, and 30 °C/48 h. Samples for the WDA were taken every four hours (30 °C) and once a day (4 °C, 8 °C and 12 °C), whereby the cell counts of LAB (MRS, 3 d, 30 °C) and the anti-*Listeria* activity in a WDA were examined. The stability of the anti-*Listeria* substance of *Lc. carnosum* DH25 was determined after the incubation of the CFS at 4 °C, 8 °C, 12 °C, and 30 °C, followed by a WDA, as described in 2.3.2. The experimental procedure is as follows: 30 mL of the culture of *Lc. carnosum* DH25 was prepared, and samples were taken every four hours (24 °C and 30 °C) or once a day (4 °C, 8 °C, and 12 °C), and the pH, cell counts of LAB (MRS, 3 d, 30 °C) and anti-*Listeria* activity (WDA) were determined. To verify the stability of the anti-*Listeria* substance, CFS was collected from a 48 h culture (30 °C) of *Lc. carnosum* DH25 was incubated at 4 °C, 8 °C, and 12 °C for 24 h, at 60 °C and 100 °C for 30 min, and at 120 °C for 15 min. In addition, the pH of the CFS was adjusted to pH 2, 3, 4, 6, 8, and 10 and incubated at room temperature for two hours before it was re-set to pH 7. A WDA was performed, including all heat- and pH-treated CFS, and compared to the non-treated CFS. The assays were performed with three independent CFSs of each LAB.

#### 2.6.2. Size Estimation of the Proteinacoeus Anti-*Listeria* Substance of *Lc. carnosum* DH25

The CFS of *Lc. carnosum* DH25 (26 h, 30 °C) was concentrated via protein size exclusion. First, it was filtered via ultra-filtration (PALL Corporation, Basel, Switzerland, Minimate^TM^ EVO TFF System, OAPMPUNV) using a 10kDA membrane filter (PALL Corporation, Minimate^TM^ TFF Capsules, PN OA010C12). The filtrate was concentrated (1:10) using a 1kDA membrane filter (PALL Corporation, Minimate^TM^ TFF Capsules PN, OA001C12). Further analyses proceeded with the retentate (with a protein size between 1 kDa and 10 kDa). Sodium Dodecyl Sulphate-PolyAcrylamide Gel Electrophoresis (SDS-PAGE) was utilized using a gradient Mini-PROTEAN TGX Gel with 4–20% acrylamide (BIO RAD, Marnes-la-Coquette, France) and the Dual Xtra Prestained Protein Standard (BIO RAD, Marnes-la-Coquette, France). The bacteriocin activity gel assay was utilized as described by Miescher et al. (2000) [18] with some modifications. Samples were loaded identically on two halves of a gel, which was then cut into two parts after the electrophoresis. As a positive control, 0.1% of bovine serum albumin (BSA) (A7906, Sigma-Aldrich^®^, Burlington, MA, USA) was loaded on SDS-PAGE. The protein bands on one half were stained with the PageBlue^TM^ Protein Staining Solution (Thermo Fisher Scientific Inc., Basel, Switzerland). For renaturation, the other half of the gel was soaked in 2.5% of Triton X-100 for 1 h at room temperature with gentle agitation and then washed in distilled water for 4 h. This gel was placed on a BHI agar plate and overlaid with BHI soft agar (0.8% *w*/*v*), inoculated with 4 log CFU/mL *L. monocytogenes* SLCC 27555 and incubated for 24 h at 37 °C.

### 2.7. Safety Assessement of Lc. carnosum DH25

#### 2.7.1. Phenotypic Detection of Biogenic Amine Formation by the Decarboxylase Assay

An undesired production of biogenic amines (tyramine, histamine, putrescine, and cadaverine) was assessed using decarboxylase agar, described by Bover-Cid and Holzapfel (1999) [19]. The LAB strains were sub-cultured twice in decarboxylase broth supplemented with the corresponding amino acid precursor tyramine, histamine, putrescine, or cadaverine (1 g/L) (all obtained from Carl Roth) and incubated at 37 °C for 24 h. 1 µL of each culture was spotted onto decarboxylase agar plates and incubated at 37 °C for 4 days. For histamine, putrescine, and cadaverine, a purple halo on the agar plate was interpreted as a positive reaction, and for tyramine, a clear halo was interpreted as a positive reaction.

#### 2.7.2. Genome Sequencing of *Lc. carnosum* DH25

The genomic DNA was isolated using a lysozyme-based cell wall digestion step and the Wizard genomic DNA purification kit (Promega, Dübendorf, Switzerland). Whole genome sequencing was performed on a MiniSeq sequencer (Illumina, San Diego, CA, USA) as described previously by Stevens et al. (2019) [20]. The reads passed the standard quality checks using the software package FastQC 0.11.7 (Babraham Bioinformatics, Cambridge, UK) and were assembled using the Spades 3.13.1-based software Shovill 1. 1.0 [21,22] using default settings. The assembly was filtered for contigs > 500 bp.

Genes were identified and annotated using prokka [23]. Species were confirmed using Speciesfinder [24]. Antimicrobial resistance genes were identified using the Resistance Gene Identifier (RGI) 4.2.2 and database version 3.2.5 [25], downloaded in September 2020. Virulence genes were identified by a bi-directional-best-hit analysis with diamond [26] with a cut-off of 70% identity, using the proteome and the core protein dataset A from the virulence factor database [27] as input. Bacteriocin genes were identified using Bagel 4 [28].

### 2.8. Statistical Analysis

All meat model and raw sausage challenge tests, as well as the measurements of pH- and a_w_ values, were performed in triplicates. The mean value and the standard derivation were calculated with Excel (Microsoft^®^ Excel^®^ für Microsoft 365 MSO (Version 2302 Build 16.0.16130.20848) 64 Bit).

## 3. Results

### 3.1. Isolation and Identification of LAB Strains

In total, 274 LAB strains were isolated from 30 meat and fish products. MALDI-TOF MS identification of 51 isolates, that showed the most promising anti-*Listeria* activity in agar spot assays, revealed species-level identification of: *Latilactobacillus sakei* (37), *Lactiplantibacillus plantarum* (7), *Latilactobacillus curvatus* (3), *Lacticaseibacillus paracasei* (1), *Leuconostoc citreum* (1), *Leuconostoc carnosum* (1), and not identified (1) (Table 3).

### 3.2. Screening of Anti-Listeria Activity

In total, 51 of the 274 isolated and identified LAB strains showed inhibition against at least one of three tested *L. monocytogenes* strains, representing the three serovars 1/2a, 1/2b, 4b, at 8 °C, 15 °C, 25 °C, and 37 °C in the agar spot assay. Of the 51 LAB strains, 35 showed anti-*Listeria* activity in a WDA against at least one of the three tested *L. monocytogenes* strains, i.e., *Latilactobacillus sakei* (25), *Lactiplantibacillus plantarum* (5), *Latilactobacillus curvatus* (2), *Lacticaseibacillus paracasei* (1), *Leuconostoc carnosum* (1), and not identified (1) (Table 3).

### 3.3. Sensitivity of Anti-Listeria Active Substances to Proteolytic Enzymes and the Presence of Structural Genes of Known Bacteriocins

In total, 31 out of the 35 isolates showing anti-*Listeria* activity in the WDA were screened for their sensitivity to proteolytic enzymes in a second WDA, where all 31 isolates showed a decreased inhibition potential when proteolytic enzymes were present; *Latilactobacillus sakei* (21), *Lactiplantibacillus plantarum* (5), *Latilactobacillus curvatus* (2), *Lacticaseibacillus paracasei* (1), *Leuconostoc carnosum* (1), and not identified (1) (Table 3). In a PCR assay for structural genes of known bacteriocins, 40 of the 51 tested LAB strains revealed a fragment at the respective size of at least one of the tested bacteriocins. The most commonly identified structural genes were those responsible for encoding the following bacteriocins: sakacins (39) leading the list, and afterward plantaracins (32) and curvacins (4). Pediocin PA-1/AcH, however, was only found in 2 strains of which *L. plantarum* strains (7) revealed pediocin PA-1/AcH, plantaricin A, KJ, S, and W, *L. paracasei* (1) plantaricin EF and W, *L. curvatus* curvacin A and sakacin Q, *L. sakei* (27) curvacin A, sakacin P and Q, and plantaricin W, *L. citreum* (1) curvacin A, and *Lc. carnosum* plantaricin A, S, and W (Table 3).

### 3.4. Challenge Test with a Meat Model and Raw Sausages

**Figure 1:**
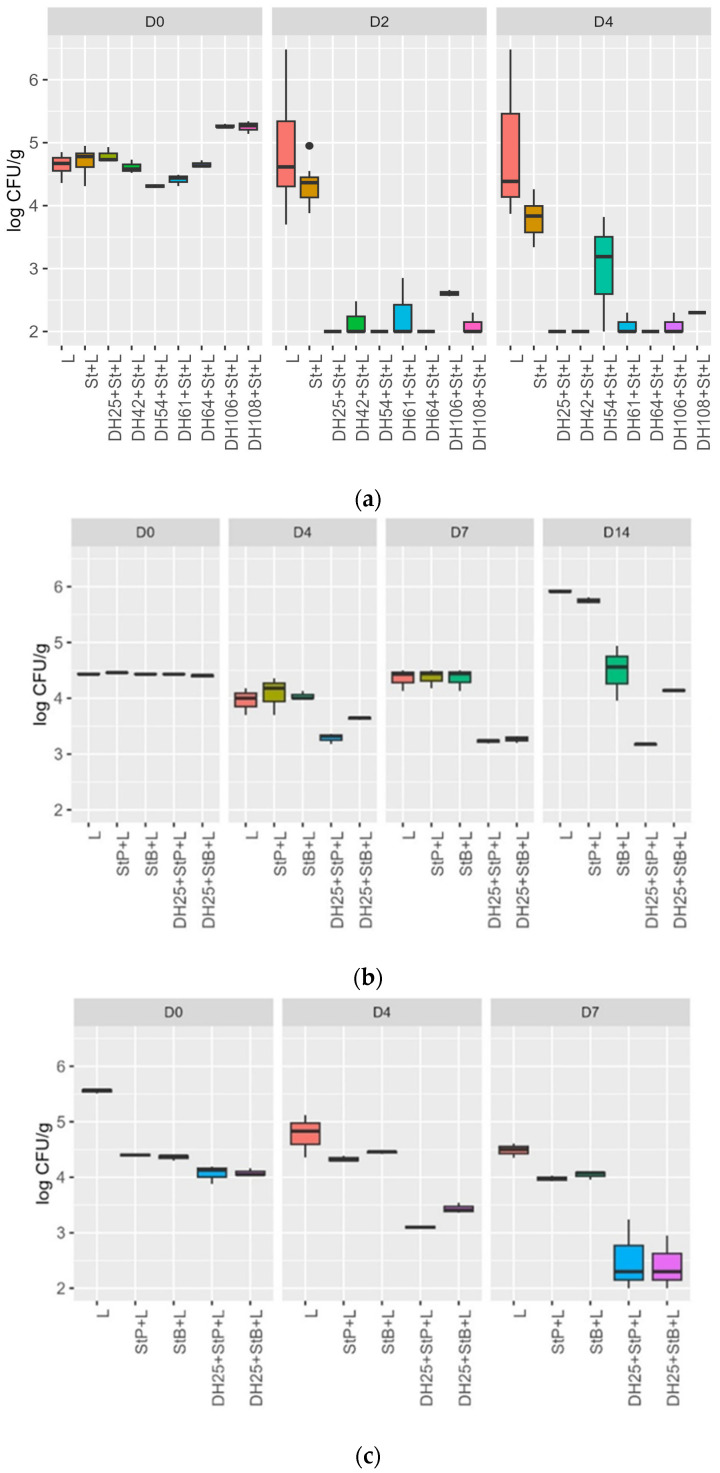
(**a**): Log CFU/g of *Listeria monocytogenes* N18-440 during a meat model challenge test, simulating the ripening of a raw sausage on days 0, 2, and 4. Seven samples were inoculated with the following: L = *Listeria monocytogenes*, St+L = starter culture + *Listeria monocytogenes*, DH25, DH42, DH54, DH61, DH64, DH106, DH108+St+L= LAB strain + starter culture + *Listeria monocytogenes*; as the starter culture, BessaStart (Moguntia Food Group, Gossau, Switzerland) was used. (**b**): Log CFU/g of *Listeria monocytogenes* N18-440 in a meat model challenge test for 14 days at 8 °C on day 0, day 4, day 7, and day 14. Five samples were inoculated with the following: L = *Listeria monocytogenes*, StP+L = Starter culture PrestoStart + *Listeria monocytogenes*, StB+L = Starter culture BessaStart + *Listeria monocytogenes*, DH25+StB+L = LAB strain + Starter culture BessaStart + *Listeria monocytogenes*, DH25+StP+L = LAB strain + Starter culture PrestoStart + *Listeria monocytogenes*. (**c**): CFU/g of *Listeria monocytogenes* N18-440 in a meat model challenge test for 7 days at 12 °C on day 0, day 4, and day 7. Five samples were inoculated with the following: L = *Listeria monocytogenes*, StP+L = Starter culture PrestoStart + *Listeria monocytogenes*, StB+L = Starter culture BessaStart + *Listeria monocytogenes*, DH25+StB+L = LAB strain + Starter culture BessaStart + *Listeria monocytogenes*, DH25+StP+L = LAB strain + Starter culture PrestoStart + *Listeria monocytogenes*.

In total, 7 LAB strains (*Lc. carnosum* DH25, *L. sakei* DH42, *L. sakei* DH54, *L. sakei* DH61, *L. sakei* DH64, *L. plantarum* DH106, and *L. plantarum* DH108) out of 36 screened LAB strains (36 strains marked in bold Table 3) tested in a meat model challenge test, simulating the ripening of a raw sausage for 4 days, showed an inhibition of the growth of *L. monocytogenes* N18-440, as shown in Figure 1a. The control samples inoculated with only *L. monocytogenes* (L) and the *L. monocytogenes* + starter culture BessaStart (St+L) obtained from Moguntia Food Group (Gossau, Switzerland) showed no change in the growth of *L. monocytogenes* N18-440, whereas the samples inoculated with an anti-*Listeria* LAB strain, starter culture, and *L. monocytogenes* N18-440 (DH+L+St) showed a decrease of 2.79 ± 0.1 (*Lc. carnosum* DH25), 2.66 ± 0.06 (*L. plantarum* DH106), 3.15 ± 0.15 (*L. plantarum* DH108), 2.45 ± 0.3 (*L. sakei* DH42), 2.13 ± 0.47 (*L. sakei* DH61), 2.65 ± 0.05 (*L. sakei* DH64), and 2.31 ± 0.02 (*L. sakei* DH54) log cfu/g *L. monocytogenes* after 2 days of incubation. In samples DH25+St+L, DH54+St+L, and DH64+St+L, the log CFU/g *L. monocytogenes* was below the detection limit (<2 log CFU/g) at day 2, and in sample DH42+St+L on day 4. The sample L showed a slight increase in growth from 4.64 ± 0.15 log CFU/g (D0) to 4.88 ± 0.99 log CFU/g (D4) and the sample St+L showed a decrease from D0 to D4 of 4.71 ± 0.21 log CFU/g to 3.80 ± 0.28 log CFU/g (Figure 1a).

The a_w_ values of 0.99 ± 0.00 measured on D0, D2, and D4 did not change during the time–temperature profile of the meat model challenge test, simulating the ripening of raw sausage (the challenge test is shown in Figure 1a). The pH values in all samples decreased between 0.49 and 0.90 from D0 to D2, and values increased between 0.10 and 0.45 from D2 to D4 (Table 4).

Log CFU/g *L. monocytogenes* of a meat model challenge test for 14 days at 8 °C, inoculated with *L. monocytogenes* N18-440, the starter cultures BessaStart and PrestoStart and protective culture *L. carnosum* DH25 are shown in Figure 1b. The samples L and StP + L showed a growth of 1.48 log CFU/g and 1.29 log CFU/g over 14 days of incubation, respectively. The sample StB+L showed no significant change in log CFU/g between day 0 and day 14. By contrast, the two samples, DH25+StP+L and DH25+Stb+L, showed a decrease of 0.26 log CFU/g and 1.26 log CFU/g for 14 days at 8 °C, respectively (Figure 1b). No significant change in a_w_ values was recognized during incubation for 14 days at 8 °C; pH values in all samples decreased between 0.5 and 0.69 from D0 to D4 and further increased between 0.69 and 2.00 from D4 to D14 (Table 5).

The results of the meat model challenge test for 7 days at 12 °C, inoculated with *L. monocytogenes* N18-440, the starter cultures BessaStart and PrestoStart, and protective culture *L. carnosum* DH25 are shown in Figure 1c. The two samples, DH25+StP+L and DH25+StB+L, showed a decrease of 1.66 log CFU/g and 1.57 log CFU/g of *L. monocytogenes* from D0 to D7. The control sample L showed a decrease of 1.06 log CFU/g from D0 to D7. The other two control samples, StP+L and StB+L, showed no significant change in log CFU/g *L. monocytogenes* over the 7-day challenge test (Figure 1c). No significant change in a_w_ values during incubation for 14 days at 12 °C was recognized, and pH values in all samples decreased between 0.62 and 1.07 from D0 to D7 (Table 6).

**Figure 2:**
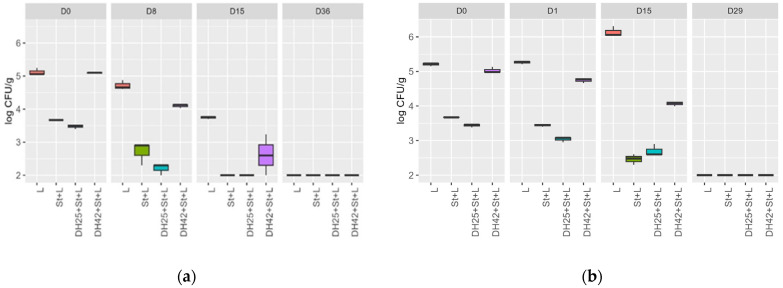
Log CFU/g of *Listeria monocytogenes* N18-440 in a challenge test during the ripening and storage of a raw sausage for (**a**) Salami on day 0, day 8, day 15, and day 36 and (**b**) Mettwurst on day 0, day 1 day 15 and day 29. Four samples were inoculated with the following: L = *Listeria monocytogenes*, St+L = Starter culture + *Listeria monocytogenes*, DH25+St+L and DH42+St+L = LAB strain + Starter culture + *Listeria monocytogenes*; as the starter culture, PrestoStart (Moguntia Food Group, Gossau, Switzerland) was used.

The growth progression of the *L. monocytogenes* strain N18-440 in a challenge test was tracked during the ripening phase of raw sausage (day 0 to day 15) and the subsequent storage period (day 15 to day 36) in samples with and without the anti-*Listeria* active LAB strains *Lc. carnosum* DH25 and *L. sakei* DH42 and the presence of a starter culture for all strains (Figure 2a). All samples exhibited a reduction in the log CFU/g of *L. monocytogenes* during ripening and continued decreasing during storage. In the samples, L+ST, DH25+St+L, and DH42+St+L, the log CFU/g of *L. monocytogenes* fell below the detection threshold (<2 log CFU/g) by the end of ripening (day 15). Following storage (day 36), *L. monocytogenes* was undetectable in all samples. In a separate raw sausage challenge test during the ripening (day 0 to day 1) and storage (day 2 to day 29) of Mettwurst, the log CFU/g of *L. monocytogenes* was monitored in similar samples, i.e., with and without anti-*Liseria the* active LAB strains *Lc. carnosum* DH25 and *L. sakei* DH42 and the presence of a starter culture for all strains (Figure 2b). After a brief ripening period of only 28 h, no significant change in the log CFU/g of *L. monocytogenes* was observed in any sample. Throughout storage (day 15 to day 0), sample L displayed a 0.93 log CFU/g increase. Conversely, all other samples demonstrated a decrease of 0.73 log CFU/g (DH25+St+L), 0.96 log CFU/g (DH42+St+L), and 1.21 log CFU/g (St+L) for the *L. monocytogenes* during storage (day 15 to day 0). After 34 days of storage, *L. monocytogenes* fell below the detection limit (<2 log CFU/g) in all samples. There were no significant changes in a_w_ values during both raw sausage challenge tests for Salami and Mettwurst. The initial pH values for both Salami and Mettwurst samples ranged between 5.53 and 5.58 (Table 7). In the Mettwurst samples, pH values decreased during the ripening phase and storage (D0 to D29), with only two samples (St+L and L) exhibiting a slight increase by the end of storage (D15 to D29). Conversely, the pH values for Salami decreased during the ripening phase and early storage (D0 to D15), followed by an increase at the end of storage (D15 to D36) for all samples (Table 7).

### 3.5. Growth-Dependent Production of Anti-Listeria Metabolites at Different Temperatures and Temperature and pH Stability of CFS

**Figure 3:**
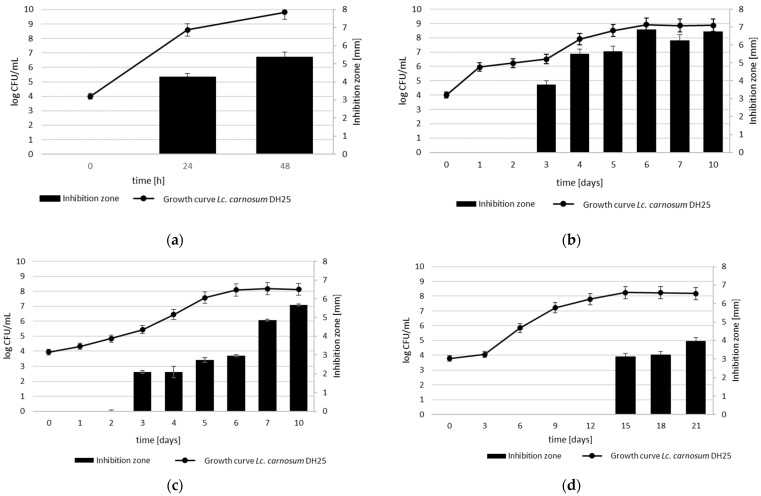
Growth curves of *Lc. carnosum* DH25 in MSM+ broth at 30 °C (**a**), 12 °C (**b**), 8 °C (**c**), and 4 °C (**d**) and inhibition zones against *L. monocytogenes* N18-440 (the radius was measured from the edge of the well to the edge of the inhibition zone in mm of corresponding CFS determined by a WDA; mean values with standard deviation (n = 3).

During growth in MSM+ broth at 30 °C *Lc. carnosum* DH25 showed anti-*Listeria* activity after 24 h and after 48 h with inhibition zones in the WDA at 4.5 mm and 5.5 mm, respectively (the radius was measured from the edge of the well to the edge of the zone of inhibition) against *L. monocytogenes* N18-220 (Figure 3a). The growth of *Lc. carnosum* DH25 in MSM+ broth at 8 °C (Figure 3c) and 12 °C (Figure 3b), respectively, revealed an anti-*Listeria* activity that was first visible after three days, reaching its maximum of 5.7 mm and 6.8 mm, respectively, after 10 days. At 4 °C, the anti-*Listeria* activity of *Lc. carnosum* DH25 was only visible on day 15, and a maximum zone of inhibition of 4 mm was reached on day 21 (Figure 3d).

**Figure 4:**
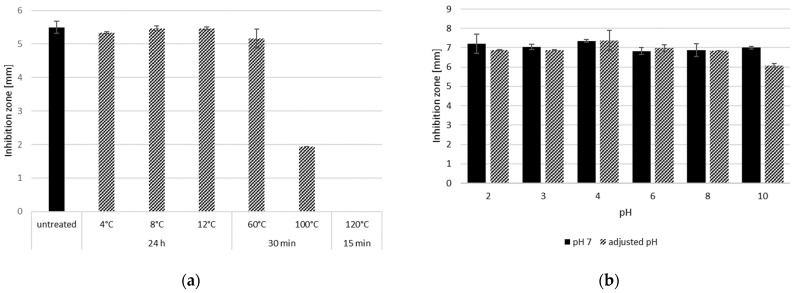
Inhibition zones against *L. monocytogenes* N18-440 (the radius was measured from the edge of the well to the edge of the zone of inhibition in mm) of heat-treated CFS (24 h at 4 °C, 8 °C, and 12 °C; 30 min at 60 °C and 100 °C; 15 min at 120 °C) compared to non-treated CFS in a WDA; mean values with standard deviation (n = 3) (**a**). Inhibition zones (radius measured from edge of the well to the edge of the zone of inhibition in mm) of CFS with pH adjusted to 2, 3, 4, 6, 8, and 10 and incubated for two hours at room temperature before being re-adjusted to pH 7, compared to CFS at pH 7 in a WDA; mean values with standard deviation (n = 2) (**b**).

Incubation of the CFS obtained from a culture of *Lc. carnosum* DH25 (48 h at 30 °C in MSM+ broth), at 4 °C, 8 °C, 12 °C, and 60 °C compared to non-treated CFS did not show a reduced ability to inhibit *L. monocytogenes* when analyzing the zone of inhibition. The incubation of the CFS at 100 °C (30 min) led to a reduction in the zone of inhibition from 5.5 mm to 2 mm and at 120 °C (15 min) to the complete loss of the zone of inhibition (Figure 4a). A pH treatment from pH 2 to 10 had no effect on the anti-*Listeria* activity in CFS of *Lc. carnosum* DH25 (Figure 4b).

### 3.6. Characterization of the Proteinaocus Anti-Listeria Metabolites Produced by Lc. carnosum DH25

**Figure 5:**
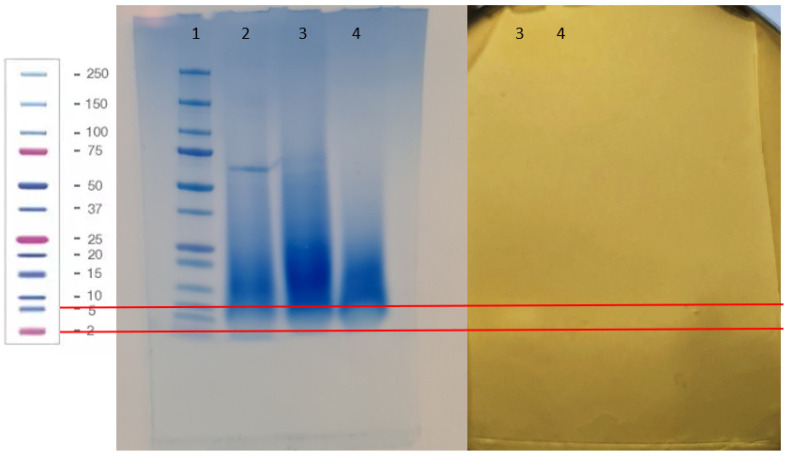
SDS-PAGE (PageBlue^TM^ Protein Staining Solution-stained gel) and bacteriocin gel activity assay (gel overlaid with BHI soft agar containing *L. monocytogenes* N18-440). (1) Dual Xtra Prestained Protein Standard (marker) (2) Positive control, 0.1% BSA (3) CFS *Lc. Carnosum* DH25 (26 h at 30 °C) 1–10 kDa (4) CFS *Lc. Carnosum* DH25 (26 h at 30 °C) ≥ 10 kDa. The red line indicates the area between the 2 and 5 kDA marker.

In an SDS-PAGE, the CFS of *Lc. carnosum* DH25 (26 h at 30 °C), filtered by ultrafiltration (1–10 kDa and ≥ 10 kDa), did not show clear bands (Figure 5; 3, 4), whereas the positive control (0.1% BSA) showed a strong band at approximately 66 kDa. The identical second half of the gel was overlayed with BHI soft agar inoculated with *L. monocytogenes* N18-440 and incubated at 37 °C for 24 h (bacteriocin gel activity assay) and revealed a bright band for *Lc. carnosum* DH25 for the CFS 1-10 kDA filtrate, between the 2 and 5 kDa marker, suggesting the production of a bacteriocin (Figure 5).

### 3.7. Safety Assessment by an Evaluation of Biogenic Amine Formation and Genome Sequencing of Lc. Carnosum DH25

In the frame of a preliminary screening safety assessment, 7 out of 51 LAB were detected to form thyramine using a decarboxylase assay and were, therefore, excluded from further experiments in this study. The formation of the biogenic amine putrescine, histamine, and cadaverine was not detected. *Lc. carnosum* DH25 did not show the formation of any of the tested biogenic amines (Table 3).

Analysis of the genome sequence of *Lc. carnosum* DH25 confirmed the MALDI-TOF MS identification and showed the absence of antibiotic-resistance genes. Only 5 potential virulence factors were identified with an elongation factor, TuEF-Tu, eno-phosphopyruvate hydratase, and 3 genes of the *rmblBADC* operon involved in polysaccharide production [29]. No toxins or factors involved in adhesion and invasion were found. A search for bacteriocin revealed the presence of the structural genes for the production of the bacteriocin LeucocinB-Ta11a in the genome of *Lc. carnosum* DH25. 

## 4. Discussion

In this study, 274 LABs were isolated from 30 different meat and fish products collected all over Switzerland, of which 51 were identified using MALDI-TOF MS. The LAB species most frequently identified were *Latilactobacillus sakei* (38); additionally, the following species could also be identified: *Lactiplantibacillus plantarum* (7), *Latilactobacillus curvatus* (3), *Lacticaseibacillus paracasei* (1), *Leuconostoc citreum* (1), and *Leuconostoc carnosum* (1). The isolation of a high number of *Latilactobacillus sakei* concurs with previous studies in which this species was reported in fermented sausage and meat products in general. The other species isolated in this study are also known to be found in the autochthonal flora of meat and fish products [30,31,32,33].

In total, 51 isolates showed anti-*Listeria* activity against at least one of three tested *L. monocytogenes* strains at 8 °C, 15 °C, 25 °C, and 37 °C. Additionally, 39 isolates showed anti-*Listeria* activity in a WDA, of which 31 revealed sensitivity to proteolytic enzymes. The sensitivity to proteolytic enzymes indicates that the CFSs used for the WDA and obtained after growth in a meat simulation medium (MSM+ broth) contained proteinaceous metabolites released into the media by the microbial strains. The anti-*Listeria* activity detected in the agar spot assay and WDA is possibly due to the production of these proteinaceous metabolites. Bacteriocins are known proteinaceous metabolites of LABs that often show anti-*Listeria* activity; in particular, class II bacteriocins are known for their potent anti-*Listeria* activity [34,35,36,37]. Bacteriocins are categorized into class I, class II (IIa, IIb, and IIc), and class III according to their biochemical and genetic properties. All the bacteriocins screened in the PCR approach used in this study belonged to class II. Class II bacteriocins are small (<10 kDA), and heat-stable peptides are known to inhibit the growth of Gram-positive food pathogens such as *L. monocytogenes* [38]. It can, therefore, be concluded that the proteinaceous metabolites of the isolates from this study are most likely bacteriocins. Proteinaceous inhibitors against *Listeria* other than bacteriocins are not known [39,40,41]. The PCR screening for the presence of structural genes of known bacteriocins supports the statement that the anti-*Listeria* activity is based on the production of bacteriocins. In total, 40 out of 51 LAB isolates showed a positive PCR reaction for at least one of the tested structural genes; pediocin AcH/PA-1 (5 strains), plantaricin A (7 strains), plantaricin EF (8 strains), plantaricin JK (2 strains), plantaricin S (6 strains), plantaricin W (10 strains), curvacin A (4 strains), sakacin G (16 strains), sakacin Q (13 strains and sakacin P (10 strains).

To verify the anti-*Listeria* activity of the isolated LAB in a meat-like environment, a meat model challenge test, simulating the ripening and storage of raw sausage at fast motion, and a raw sausage challenge test (Salami and Mettwurst) was set up and included the commercial starter cultures BessaStart and PrestoStart (Moguntia Food Group, Gossau, Switzerland). In total, 7 out of the 36 screened LAB strains tested in the meat model challenge test showed an inhibition of the growth of *L. monocytogenes* N18-440, between 2.13 and 3.15 log CFU/g during the 4 days of the challenge test. Compared to control sample L, which showed a slight increase of 0.24 log CFU/g, sample L+St showed only a slight decrease of −0.91 log CFU/g. It is known from previous studies using a similar meat model system that *L. monocytogenes* can grow in meat models despite the addition of spices, salt, and nitrite [42,43,44]. The starter culture BessaStart used in this meat model challenge test is known for mild acidification; this might explain the slight decrease in log CFU/g *L. monocytogenes* in the sample L+St; the pH value of this sample, which dropped from 5.74 to 4.91 (D0 to D2) confirms this assumption, whereas in sample L the pH only dropped to 5.06 at day 2. In all samples, the pH values decreased between 0.49 and 0.90 from D0 to D2, and values increased between 0.10 and 0.45 from D2 to D4; this behavior was also observed by Degenhardt & Sant’Anna (2007) [5], Gao et al. (2014) [35], and Thévenot et al. (2005) [45]. The pH reduction at the beginning of the challenge test could be attributed to the formation of acids due to the added lactic acid bacteria or those already present in the autochthonous flora of the meat, while the subsequent increase may be caused by the proteolysis and lipolysis carried out by yeast [5,45,46]. The study of Thévenet et al. (2005) [45] showed initial pH values in French sausages from 5.85 to 5.94 and a drop in pH on day 5 to 5.0, whereas, in this study, the initial pH values in the meat model were between 5.68 and 5.83 and dropped to pH 5 in two samples and in all other samples below pH 5 on day 2. However, it needs to be mentioned that the incubation temperature in this study ranged from 18 to 24 °C, which is higher than the one used by Thévenet et al. (2005) [45] at 10 to 15 °C. The a_w_ values remained at an initial value of 0.99 during the whole challenge test, most probably due to the shortened simulation of the ripening of a raw sausage at only 4 days without a drying or maturation step. Sample L+St+DH25 showed a decrease of −2.79 ± 0.1 log/CFU *L. monocytogenes* and L+St+DH42 −2.45 ± 0.3 after 4 days of incubation. The strains *Lc. carnosum* DH25 and *L. sakei* DH42 were further evaluated in a raw sausage challenge test and *Lc. carnosum* DH25 was also evaluated in a meat model storage challenge test at 12 °C and 8 °C. In the raw sausage challenge test (Salami), in the sample L+St+DH25, the log CFU/g *L. monocytogenes* was below the detection limit (<2 log CFU/g) at the end of ripening whereas in the samples, L, L+St, and L+St+DH42 log CFU/g *L. monocytogenes* was below the detection limit (<2 log CFU/g) only after storage. Probably due to the short ripening period of only 28 h in the second raw sausage challenge test (Mettwurst), none of the samples showed a decrease in log CFU/g *L. monocytogenes* during the ripening. Nevertheless, *L. monocytogenes* numbers decreased during the storage of the Mettwurst in all samples (0.73 log CFU/g DH25+St+L; 0.96 log CFU/g DH42+St+L; 1.21 log CFU/g St+L) except for the control sample L where an increase of 0.93 log CFU/g occurred. The capacity of *L. monocytogenes* to proliferate in raw sausage, even under refrigerated conditions, has been previously documented by various investigations [47,48,49,50,51]. For instance, Luo et al. (2015) [52] conducted an experiment wherein ready-to-eat ham and sausages were inoculated with an initial concentration of 3.05 log CFU/g *L. monocytogenes*, leading to an ultimate cell density exceeding 8 log CFU/g. In the present study, elevated initial concentrations (5 log CFU/g) were employed, and the maximum cell densities observed in the control samples were 6 log CFU/g in both the meat model and the raw sausage (Mettwurst) challenge test. Parallel findings were reported by Hugas et al. (1995) [48], who observed comparable results when inoculating a meat model system with *L. monocytogenes* at an initial concentration of 4 log CFU/g, reaching 5 log CFU/g by day 10.

At the end of the storage in all samples, *L. monocytogenes* numbers were below the detection limit of 2 log CFU/g. During the meat model challenge test at 8 °C, the samples L+StB+DH25 and L+StP+DH25 showed a decrease of 0.26 and 1.26 log CFU/g, whereas the control samples (L, L+StP, L+STB) showed an increase of 1.48 and 1.29 log CFU/g over 14 days of storage. The meat model challenge test at 12 °C showed similar results; samples L+StB+DH25 and L+StP+DH25 showed a decrease of 1.66 log and 1.57 log CFU/g *L. monocytogenes* and the control samples did not show a change in log CFU/g *L. monocytogenes*. The pH value curve for the raw sausage and storage challenge tests compared to the meat model challenge test are comparable; after an initial decrease, the pH-value increased again slightly towards the end of the challenge tests. According to EC Reg. 2073/2005 [53], the growth of *L. monocytogenes* is not supported in ready-to-eat foods such as fermented sausages if pH ≤ 4.4 and a_w_ ≤ 0.92 or pH ≤ 5.0 and a_w_ ≤ 0.94. In none of the challenge tests carried out in this study were the a_w_- and pH-values low enough to be responsible for *Listeria* inhibition. Since the *Listeria* cell counts were stable or even increased in the absence of anti-*Listeria* LAB strains, the added spices, as well as the salt and nitrite, could also be ruled out for inhibition. The inhibition of *L. monocytogenes* in all challenge tests performed in this study could, therefore, mainly be attributed to the anti-*Listeria* activity of bacteriocin-like substances formed via the addition of LAB strains. Several studies have also proven that bacteriocin-producing strains such as *L. curvatus*, *Lc. carnosum*, *L. sakei*. and *L. plantarum* is able to control the growth of the food pathogen *L. monocytogenes* in fermented sausage during the fermentation as well as at lower temperatures up to 2 °C during the storage of sausages [34,47,49,50,54]. Castellano & Vignolo (2006) [34] investigated the inhibition of the growth of *Listeria innocua* via the bacteriocin-producing *Lactobacillus curvatus* CRL705 in a challenge test with vacuum-packed meat stored at 2 °C for 36 days. *Listeria* was able to grow in the control samples up to 3 log CFU/g, whereas in the samples inoculated with *L. curvatus* CRL705, cell counts of *L. innocua* remained stable during the challenge test.

The anti-*Listeria* substances of *Lc. carnosum* DH25, the most promising strain in this study, was tested for its temperature and pH stability. The incubation of CFSs (30 °C/48 h in MSM+ broth) at 4 °C, 8 °C, 12 °C, and 60 °C did not show a reduced zone of inhibition in a WDA; incubation at 100 °C for 30 min led to reduction and incubation at 120 °C for 15 min to a complete loss of the zone of inhibition. The pH treatment of the CFS (pH 2 to 10) had no effect on the anti-*Listeria* activity of the CFS. Kumar et al., 2016 [55] conducted a similar study with antimicrobials of an *L. plantarum*, where the anti-*Listeria* activity remained completely stable even at 121 °C, but at a pH above 6 showed a loss in activity of up to 60%. A study by Castro et al., 2010 [49] demonstrated comparable results in their study, wherein the extracellular extracts of *L. sakei* maintained antimicrobial activity even after heat treatment. The antimicrobial activity showed no decline when subjected to incubation at temperatures of up to 100 °C for 15 min, storage at 5 °C for 21 days, and three freeze–thaw cycles.

Despite no specific band on the SDS-PAGE, the bacteriocin gel activity assay showed slight anti-*Listeria* activity between the 2 and 5 kDa marker shown by a bright band. Daba et al., 2022 [56] observed similar findings, where no specific band on the SDS-PAGE appeared, but an antimicrobial activity was observed nevertheless. Despite the likely presence of only a minimal concentration of the anti-*Listeria* substance in the CFS of *Lc. carnosum* DH25, its efficacy was still demonstrated. The structural gene for the production of the bacteriocin leucocin B-Ta11a is present in the genome of *Lc. carnosum* DH25; it belongs to class IId bacteriocins with a size of 3.48 kDa [50,57]. Budde et al. (2003) [50] isolated *Lc. carnosum* 4010 from vacuum-packed meat; the strain showed anti-*Listeria* activity, which was caused by leucocin A-4010 and leucocin B-4010, in which leucocin B-4010 was identical to leucocin B-Ta11a. It is very likely that the anti-*Listeria* activity seen in this bacteriocin gel activity assay and in the other experiments of this study either results from a bacteriocin identical to leucocin B-Ta11a or a novel bacteriocin expressed by *Lc. carnosum* DH25.

The genome sequencing of *Lc. carnosum* DH25 confirmed the identification of MALDI-TOF MS and showed no presence of any antibiotic-resistance genes. Furthermore, only five virulence factors (VFs) were identified. In the closely related genus, *Streptococcus,* between 20 and 40 VFs are found in pathogenic *Streptococcus* strains and 6 in the food-grade *Streptococcus thermophilus* strain LMD-9 [27]. In addition, the VFs found were involved in elongation and sugar metabolism and were likely niche factors rather than VFs [58]. Moreover, *Lc. carnosus* DSM 32756 has GRAS status [59] and approval for *Lc. carnosum* DH25, which can be used in food as a protective culture, will not be a problem.

## 5. Conclusions

In conclusion, this study underscores the critical need to control *L. monocytogenes* growth in food to prevent listeriosis, a severe infection with high mortality rates. This research identified 51 LAB isolates with anti-*Listeria* activity, primarily due to the production of bacteriocins, particularly class II bacteriocins known for their effectiveness against *L. monocytogenes*. Many of these LAB isolates demonstrated their efficacy in challenging conditions, such as meat model tests and raw sausage challenges, offering a promising solution for inhibiting *L. monocytogenes* growth. Of particular significance was the outstanding performance of *Lc. carnosum* DH25. This strain consistently demonstrated anti-*Listeria* activity across various tests, including meat model challenge tests and raw sausage challenge tests, outperforming other screened LAB strains. These findings indicate that *L. carnosum* DH25 effectively inhibits the growth of *L. monocytogenes* under meat storage conditions, even at low temperatures (8–12 °C). Notably, the anti-*Listeria* substances produced by *Lc. carnosum* DH25 showed remarkable stability at different temperatures (4–100 °C) and pH conditions (pH 2–10). The genome sequencing of *Lc. carnosum* DH25 confirmed its safety profile, making it a potential protective culture for raw sausages. This research contributes significantly to food safety, particularly in the context of preventing *L. monocytogenes* growth in raw sausages.

## Figures and Tables

**Table 1:** Meat and fish products used in this study for the isolation of LAB.

ID ^1^	Product Type ^2^	Animal Origin ^4^	Package ^5^	Aliquot Size ^6^	Growth Media ^7^
**Fish products**					
H1	Graved fish	Farmed salmon (NO)	UP, 4 °C	40 g	MRS, DMRS, ELK
H6	Fresh fish, after thawing (I) ^3^	Wild salmon (AK)	UP, 4 °C	40 g	MRS, DMRS, ELK
H7	Fresh fish, dry salted (I)	Wild salmon (AK)	UP, 4 °C	40 g	MRS, DMRS, ELK
H9	Fresh fish, salted by injection of brine (I)	Wild salmon (AK)	UP, 4 °C	40 g	MRS, DMRS, ELK
H8	Fresh fish, salted by injection of brine (I)	Farmed salmon (NO)	UP, 4 °C	40 g	MRS, DMRS, ELK
H4	Cold-smoked fish	Farmed salmon (NO)	VP, −20 °C	20 g	MRS, DMRS, ELK
H10	Cold-smoked fish	Farmed salmon (NO)	UP, 4 °C	40 g	MRS, DMRS, ELK
**Meat products**					
H2	Fermented sausage	Beef, pork (CH)	VP, RT	40 g	MRS, DMRS, ELK
H3	Fermented sausage	Elk (EU), pork (EU)	MAP, RT	20 g	MRS, DMRS, ELK
H5	Fermented sausage	Boar (CH)	UP, RT	40 g	MRS, DMRS, ELK
H11	Fresh meat, dry salted (I)	Beef (CH)	UP, 4 °C	40 g	MRS, DMRS, ELK
H12	Dry-cured meat	Ibex (CH)	VP, RT	20 g	MRS, DMRS
H13	Fermented sausage, lightly smoked	Beef, pork (CH)	VP, RT	20 g	MRS, DMRS
H14	Fresh sausage, lightly smoked	Beef, pork (CH)	VP, 4 °C	40 g	MRS, DMRS
H15	Dry-cured meat	Deer (AT)	VP, RT	20 g	MRS, DMRS
H16	Dry-cured meat	Pork (CH)	VP, RT	20 g	MRS, DMRS
H17	Fermented sausage	Chamois, pork (CH)	VP, RT	20 g	MRS, DMRS
H18	Fresh sausage (I)	Beef (CH)	UP, 4 °C	40 g	MRS, DMRS
H19	Fermented sausage	Beef (CH)	VP, RT	20 g	MRS, DMRS
H20	Fresh sausage (I)	Sheep, pork (CH)	UP, 4 °C	40 g	MRS, DMRS
H21	Fermented sausage	Sheep, pork (CH)	UP, RT	20 g	MRS, DMRS
H22	Fresh sausage with 9% beetroot (I)	Beef, pork (CH)	UP, 4 °C	40 g	MRS, DMRS
H23	Fermented sausage with 9% beetroot	Beef, pork (CH)	UP, RT	20 g	MRS, DMRS
H24	Fermented sausage	Goat (CH)	UP, RT	20 g	MRS, DMRS
H25	Fermented sausage	Deer (CH/NZ), pork (CH)	UP, RT	20 g	MRS, DMRS
H26	Fermented sausage	Pork (CH)	UP, RT	20 g	MRS, DMRS
H27	Fermented sausage	Boar (CH/NZ), pork (CH)	UP, RT	20 g	MRS, DMRS
H28	Dry-cured meat	Veal (n.i.)	UP, RT	20 g	MRS, DMRS
H29	Fermented sausage	Deer (CH/AUT), pork (CH)	UP, RT	20 g	MRS, DMRS
H30	Fermented sausage	Pheasant, pork (EU)	UP, RT	20 g	MRS, DMRS

^1^ Producer information: H1: Rageth Comestibles AG, Landquart, Switzerland; H3 and H4: IKEA Switzerland; H6–H10: Ospelt Food AG, Weite, Switzerland (Wild salmon (Sockeye) was frozen in Alaska for transport and thawed in Switzerland before further processing, farmed salmon was delivered from Norway and was stored on ice for transport); H2 and H11–H17: Naturtrocknerei Jörg Brügger & Co, Parpan, Switzerland; H18–H24: Metzgerei Beat Eggs, Reckingen, Switzerland; H25–H27 ENZ Premium, Schönholzerswilen, Switzerland; H5: Macelleria e Salumeria Delmenico, Sessa, Switzerland; H29: Alphüsli, Metzgerei Venzin, Disentis Switzerland; H28: Namli Pastirma, Istanbul, Turkey; H30: Salaisons du Val d’Allier, Langeac, France. The abbreviation “H” originally stood for “habitat”. ^2^ Graved fish: fish preserved by the addition of sugar and salt, ready to eat; fresh fish: fish, salted or unsalted but not yet smoked, not ready to eat; cold-smoked fish: fish preserved by salting and smoking, ready to eat; fresh sausage: sausage from chopped meat, cured but not yet fermented, not ready to eat; fermented sausage: sausage from chopped meat, cured and fermented, ready to eat; fresh meat: a whole piece of meat, cured but not dried, not ready to eat; dry-cured meat: a whole piece of meat, preserved by curing and drying, ready to eat. ^3^ I = intermediate product: a product sample that was taken during the production process; H06–H09: fish samples taken at different stages during the production of cold-smoked salmon; H11: meat sample taken during the production of Bündnerfleisch; H18, H20, and H22: sausage samples taken at fermentation day 0 during the production of fermented sausages. ^4^ AK = Alaska; AUT = Austria, CH = Switzerland, EU = European Union, NO = Norway; NZ = New Zealand, n.i. = no information. ^5^ RT = room temperature, UP = unpacked or wrapped in paper; VP = vacuum-packed, MAP = modified atmosphere packaging. ^6^ Sample aliquots used for enumeration of LAB; depending on the size of the product, 20 or 40 g were analyzed. ^7^ Agar media used for enumeration of LAB: MRS = De Man Rogosa Sharp agar, DMRS = modified MRS agar, ELK = Elliker Agar.

**Table 2:** Parameters for the ripening process of a raw sausage (Mettwurst and Salami).

Time [h]	Temperature [°C]	Relative Humidity [%]
28	24	94
Mettwurst: storage at 2 °C, Salami: follow further ripening steps		
14	22	90
14	20	88
12	18	86
12	16	84
12	14	82
12	14	80
216	14	76

**Table 3:** Anti-*Listeria* activity of 51 isolated LAB strains determined in an agar spot assay against 3 *L. monocytogenes* strains (ATTC 15313 serovar 1/2a, SLCC 27555 serovar 1/2b und ATCC 19115 serovar 4b) at four different temperatures (8 °C, 15 °C, 25 °C, and 37 °C). *Listeria* inhibition determined in a WDA [mm]; the sensitivity of the anti-*Listeria* substances to proteolytic enzymes; presence of structural genes of known bacteriocins determined by PCR, and the formation of biogenic amine (tyramine, histamine, putrescine, and cadaverine) ^1^.

Strain	Agar Spot Assay and WDA												Positive PCR Reaction (Primer)	Sensitivity to Proteolytic Enzymes	Biogenic Amine Formation (Tyramine, Histamine, Putrescine, and Cadaverine)
	*L. monocytogenes* ATTC 15313				*L. monocytogenes* SLCC 27555				*L. monocytogenes* ATCC 19115						
	37 °C/mm	25 °C	15 °C	8 °C	37 °C/mm	25 °C	15 °C	8 °C	37 °C/mm	25 °C	15 °C	8 °C			
***Lactiplantibacillus plantarum*** **DH2**	4.55	++++S	++++S	++++S	6.9	++++S	++++S	++++S	7.2	++++S	++++S	++++S	Ped, Pln A, PlanW, Pln	Yes	none
***Lactiplantibacillus plantarum*** **DH3**	5.1	++++S	++++S	++++S	6.95	++++S	++++S	++++S	7.5	++++S	++++S	++++S	Pln A, Pln	Yes	none
***Lactiplantibacillus plantarum*** **DH9**	3.6	++++S	++++S	++++S	6.85	++++S	++++S	++++S	7.6	++++S	++++S	++++S	Ped, Pln A, PlanS, Pln	Yes	none
***Leuconostoc carnosum* DH25**	3.5	++++S	++++S	++++S	6.25	++++S	++++S	++++S	4.85	++++S	++++S	++++S	Pln A, PlanS, PlanW	Yes	none
*Latilactobacillus curvatus* DH29	0	+++	++++S	++++S	7.55	++++	++++S	++++S	0	++	++++	++++S	CurA, Sak Q	NA	none
***Latilactobacillus sakei* DH42**	0	++++S	++++S	++++S	3.15	++++S	++++S	++++S	4.1	+++	++++S	++++S	Sak Q, Sak P	Yes	none
***Latilactobacillus sakei*** **DH45**	0	++++	++++S	++++S	1.55	+++	++++	++++S	0.6	+++	++++S	++++S	Sak Q, Sak P	Yes	none
*Latilactobacillus sakei* DH51	0	++++S	++++S	++++S	2	++++S	++++S	++++S	ND	++++	++++S	++++S	skgA2	NA	none
***Latilactobacillus sakei*** **DH54**	0	++++S	++++S	++++S	1.85	++++S	++++S	++++S	ND	++++	++++S	++++S	PlanW, skgA2	Yes	none
***Latilactobacillus sakei*** **DH61**	0	++++S	++++S	++++S	1.65	++++	++++S	++++S	ND	++++	++++S	++++S	skgA2	Yes	none
*Latilactobacillus sakei* DH64	0	++++S	++++S	++++S	1.6	++++	++++S	++++S	ND	++++	++++S	++++S	skgA2	NA	none
***Latilactobacillus sakei*** **DH80**	0	++++S	++++S	++++S	1.15	++++	++++S	++++S	ND	++++	++++S	++++S	PlanW, skgA2	NA	none
***Latilactobacillus sakei*** **DH84**	0	++++S	++++S	++++S	1.55	++++	++++S	++++S	ND	++++	++++S	++++S	skgA2	Yes	none
***Latilactobacillus sakei*** **DH85**	0	++++S	++++S	++++S	2.1	++++	++++S	++++S	3.1	++++	++++S	++++S	skgA2	Yes	none
***Latilactobacillus sakei*** **DH87**	0	+++	+++	++++	0	++	++++S	++++	0	+++	++++	++++S	Sak P, Sak Q	NA	none
***Lactiplantibacillus*** ***plantarum* DH106**	ND	++++S	++++S	ND	ND	++++S	++++S	++++S	ND	++++S	++++S	++++S	Pln A, Pln J, Pln K, PlanS, PlanW, Pln	NA	none
***Lactiplantibacillus*** ***plantarum* DH108**	ND	+++	ND	ND	ND	+++	ND	++++	NA	+++	ND	++++S	Pln A, Pln J, Pln K, PlanS, PlanW, Pln	NA	none
***Latilactobacillus sakei*** **DH126**	0	+	++++	ND	0	+	+	++++S	0	++	+++	++++	CurA, Sak P, Sak Q	NA	none
*Latilactobacillus sakei* DH134	0	+++	++++	ND	3.35	+	+	++++	0	++	++	++++	none	NA	none
***Latilactobacillus sakei*** **DH140**	0	++++S	++++S	++++S	0	++++S	++++S	++++S	0	++++S	++++S	++++S	none	NA	none
*Latilactobacillus sakei* DH150	0	++++	++++S	++++S	0	++++S	++++S	++++S	0	++++S	++++S	++++S	Sak P, Sak Q	NA	none
***Latilactobacillus sakei*** **DH151**	0	++++	++++S	++++S	0	++++S	++++S	++++S	0	++++S	++++S	++++S	Sak P, Sak Q	NA	none
***Latilactobacillus sakei*** **DH152**	0	++++	++++S	++++S	0	++++S	++++S	++++S	0	++++S	++++S	++++S	CurA, Sak P, Sak Q	NA	none
***Latilactobacillus sakei*** **DH153**	0	++++S	++++S	++++S	0	++++S	++++S	++++S	0	++++	++++S	++++S	Sak P, Sak Q	NA	none
***Latilactobacillus sakei*** **DH165**	0	++++S	++++S	++++S	0	++++	++++	++++S	0	++++	++++	++++S	Sak P, Sak Q	NA	none
***Leuconostoc citreum*** **DH173**	0	++++S	++++S	++++S	0	++++S	++++S	++++S	0	++++	++++S	++++S	CurA	NA	none
*Lactiplantibacillus plantarum* DH176	7.05	++++S	++++S	++++S	4.45	++++S	++++S	++++S	9.05	++++S	++++S	++++S	PlanS, PlanW, Pln	Yes	none
***Latilactobacillus sakei*** **DH192**	0	++++S	++++S	++++S	0	++++S	++++S	++++S	7.65	++++S	++++S	++++S	none	Yes	none
***Latilactobacillus sakei*** **DH208**	5.5	++++S	++++S	++++S	0	++++S	++++S	++++S	0	++++S	++++S	++++S	none	Yes	none
***Latilactobacillus sakei*** **DH209**	0	++++S	++++S	++++S	0	++++	++++S	++++S	0	++++S	++++S	++++S	none	NA	none
***Latilactobacillus sakei*** **DH210**	0	++++S	++++S	++++S	0	++++S	++++S	++++S	0	++++S	++++S	++++S	none	NA	none
*Latilactobacillus sakei* DH213	0	++++	++++S	++++S	0	++++S	++++S	++++S	2.8	++++	++++S	++++S	none	Yes	Tyramine
***Latilactobacillus sakei*** **DH228**	2.25	++++S	++++S	++++S	0	++++S	++++S	++++S	1.4	++++S	++++S	++++S	none	Yes	none
*Lactiplantibacillus plantarum* DH229	7.8	++++S	++++S	++++S	4.8	++++S	++++S	++++S	9.45	++++S	++++S	++++S	PlanW	Yes	Tyramine
***Latilactobacillus sakei*** **DH230**	0	++++S	++++S	++++S	4.35	++++S	++++S	++++S	8.15	++++S	++++S	++++S	skgA2	Yes	none
***Latilactobacillus sakei*** **DH231**	0	++++S	++++S	++++S	0	++++S	++++S	++++S	0	++++S	++++S	++++S	skgA2	NA	none
***Latilactobacillus sakei*** **DH232**	1.6	++++S	++++S	++++S	2.6	++++S	++++S	++++S	0	++++S	++++S	++++S	skgA2	Yes	none
***Latilactobacillus sakei*** **DH233**	0.75	++++S	++++S	++++S	2.7	++++S	++++S	++++S	0	++++S	++++S	++++S	skgA2	Yes	none
***Lactobacillus curvatus*** **DH234**	0	++++S	++++S	++++S	4	++++S	++++S	++++S	5.8	++++S	++++S	++++S	none	Yes	none
*Latilactobacillus sakei* DH235	0	++++S	++++S	++++S	9.35	++++S	++++S	++++S	0.8	++++S	++++S	++++S	none	Yes	none
*Latilactobacillus sakei* DH250	0	++++S	−	++++S	0.85	++++S	++++S	++++S	0	++++S	++++S	++++S	Sak Q	Yes	Tyramine
*Latilactobacillus curvatus* DH252	0	++++S	−	++++S	0	++++S	++++S	++++S	0	++++S	++++S	++++S	Sak Q	Yes	Tyramine
***Latilactobacillus sakei*** **DHN35**	0	++++S	++++S	++++S	0	++++S	++++S	++++S	0	++++S	++++S	++++S	Sak P, Sak Q	NA	none
***Latilactobacillus sakei*** **DHN129**	1.55	++++S	++++S	++++S	2.45	++++S	++++S	++++S	0	++++S	++++S	++++S	skgA2	Yes	none
*Latilactobacillus sakei* DHN154	1.45	++++S	++++S	++++S	2.3	++++S	++++S	++++S	0	++++S	++++S	++++S	skgA2	Yes	Tyramine
*Latilactobacillus sakei* DHN158	1.9	++++S	++++S	++++S	2.5	++++S	++++S	++++S	0	++++S	++++S	++++S	skgA2	Yes	Tyramine
***Latilactobacillus sakei*** **DHN62**	0.65	++++S	++++S	++++S	2.3	++++S	++++S	++++S	3.25	++++S	++++S	++++S	skgA2	Yes	none
***Latilactobacillus sakei*** **DHN81**	0	++++S	++++S	++++S	2.95	++++S	++++S	++++S	0	++++S	++++S	++++S	skgA2	Yes	none
*Latilactobacillus sakei* PR21-05	0	++++S	++++S	++++S	10.05	++++S	++++S	++++S	1.6	++++S	++++S	++++S	none	Yes	Tyramine
**Not identified PR21-08**	0	++++S	++++S	++++S	2.55	++++S	++++S	++++S	1	++++S	++++S	++++S	Pln	Yes	none
*Lacticaseibacillus paracasei* PR25-02	0	++++S	++++S	++++S	0	++++S	++++S	++++S	1.95	++++S	++++S	++++S	Pln, PlanW	Yes	none

^1^ Bold: Strains were screened for anti-*Listeria* activity in a meat model challenge test. Rating agar spot assay: red: 0–0.9 mm halo (−), beige: 1–2.9 mm halo (+), orange: 3–4.9 mm halo (++), yellow: 5–6.9 mm halo (+++), light green: 7–14.9 mm halo (++++), dark green: >14.9 mm halo (++++S). ND = no data, NA = not analysed.

**Table 4:** pH and a_w_ values during the time–temperature profile of a meat model challenge test with *Listeria monocytogenes* N18-440, simulating the ripening of a raw sausage on days 0, 2, and 4 (challenge test shown in Figure 1a). Seven samples were inoculated with the following: L = *Listeria monocytogenes*, St+L = starter culture + *Listeria monocytogenes*, DH25, DH42, DH54, DH61, DH64, DH106, DH108+St+L= LAB strain + starter culture + *Listeria monocytogenes*; as the starter culture, BessaStart (Moguntia Food Group, Gossau, Switzerland) was used. pH; n = 3, a_w_; n = 3.

**Sample**	**pH Ø**		
	**D0**	**D2**	**D4**
L+St+DH25	5.83 ± 0.01	4.93 ± 0.05	5.15 ± 0.03
L+St+DH42	5.76 ± 0.01	4.95 ± 0.10	5.07 ± 0.14
L+St+DH54	5.70 ± 0.02	5.01 ± 0.02	4.99 ± 0.22
L+St+DH61	5.63 ± 0.00	5.14 ± 0.06	5.59 ± 0.63
L+St+DH64	5.68 ± 0.01	4.81 ± 0.03	5.11 ± 0.13
L+St+DH106	5.73 ± 0.01	4.91 ± 0.06	5.01 ± 0.09
L+St+DH108	5.76 ± 0.04	4.89 ± 0.08	5.22 ± 0.02
L	5.77 ± 0.06	5.06 ± 0.27	5.16 ± 0.27
L+St	5.74 ± 0.10	4.91 ± 0.13	5.18 ± 0.26
	**a_w_ Ø**		
Control	0.99 ± 0.00	0.99 ± 0.00	0.99 ± 0.00

**Table 5:** pH and a_w_ values during a meat model challenge test with *Listeria monocytogenes* N18-440 for 14 days at 8 °C on days 0, 4, 7, and 14 (challenge test shown in Figure 1b). Five samples were inoculated with the following: L = *Listeria monocytogenes*, StP+L = Starter culture PrestoStart + *Listeria monocytogenes*, StB+L = Starter culture BessaStart + *Listeria monocytogenes*, DH25+StB+L = LAB strain + Starter culture BessaStart + Listeria monocytogenes, DH25+StP+L = LAB strain + Starter culture PrestoStart + *Listeria monocytogenes*; as the starter culture, BessaStart and PrestoStart (Moguntia Food Group, Gossau, Switzerland) were used. pH; n = 1, a_w_; n = 3.

**Sample**	**pH**			
	**D0**	**D4**	**D7**	**D14**
DH25+B+L	5.86	5.28	5.54	7.28
DH25+P+L	5.74	5.24	5.51	6.05
B+L	5.80	5.29	5.60	7.21
P+L	5.86	5.30	5.62	6.44
L	5.90	5.21	5.67	5.90
	**a_w_ Ø**			
Control	0.98 ± 0.01	0.99 ± 0.00	0.97 ± 0.00	0.99 ± 0.00

**Table 6:** pH and a_w_ values during a meat model challenge test with *Listeria monocytogenes* N18-440 for 7 days at 12 °C on days 0, 4, and 7 (challenge test shown in Figure 1c). Five samples were inoculated with the following: L = *Listeria monocytogenes*, StP+L = Starter culture PrestoStart + *Listeria monocytogenes*, StB+L = Starter culture BessaStart + *Listeria monocytogenes*, DH25+StB+L = LAB strain + Starter culture BessaStart + *Listeria monocytogenes*, DH25+StP+L = LAB strain + Starter culture PrestoStart + *Listeria monocytogenes*; as the starter culture, BessaStart and PrestoStart (Moguntia Food Group, Gossau, Switzerland) were used. pH; n = 1, aw; n = 3.

**Sample**	**pH**		
	**D0**	**D4**	**D7**
DH25+B+L	6.06	5.65	5.29
DH25+P+L	6.05	5.25	5.43
B+L	6.03	5.69	5.24
P+L	6.11	5.34	5.04
L	6.07	5.93	5.29
	**a_w_ Ø**		
Control	0.96 ± 0.00	0.95 ± 0.00	0.95 ± 0.00

**Table 7:** pH and a_w_ values in a challenge test with *Listeria monocytogenes* N18-440 during the ripening and storage of raw sausages, Salami and Mettwurst, on day 0, day 8, day 15, and day 36 and day 0, day 1, day 15, and day 29, respectively (challenge tests showed in Figure 2). Four samples were inoculated with the following: L = *Listeria monocytogenes*, St+L = Starter culture + *Listeria monocytogenes*, DH25+St+L and DH42+St+L = LAB strain + Starter culture + *Listeria monocytogenes*; as the starter culture, PrestoStart (Moguntia Food Group, Gossau, Switzerland) was used. pH; n = 3, a_w_; n = 3.

**Sample**	**pH Ø**					
**Salami**	**D0**	**D1**	**D8**	**D15**	**D29**	**D36**
**DH25+St+L**	5.58 ± 0.00	-	4.76 ± 0.02	4.71 ± 0.04	-	4.89 ± 0.00
**DH42+St+L**	5.53 ± 0.02	-	4.80 ± 0.00	4.62 ± 0.01	-	4.82 ± 0.02
**St+L**	5.56 ± 0.00	-	4.85 ± 0.01	4.72 ± 0.02	-	4.90 ± 0.01
**L**	5.55 ± 0.00	-	5.20 ± 0.01	4.70 ± 0.00	-	4.91 ± 0.01
**Mettwurst**						
**DH25+St+L**	5.55 ± 0.01	5.41 ± 0.00	-	4.89 ± 0.00	4.82 ± 0.01	-
**DH42+St+L**	5.56 ± 0.00	5.40 ± 0.00	-	4.87 ± 0.02	4.86 ± 0.00	-
**St+L**	5.56 ± 0.00	5.29 ± 0.01	-	4.74 ± 0.01	4.91 ± 0.00	-
**L**	5.55 ± 0.00	5.30 ± 0.01	-	4.79 ± 0.00	4.81 ± 0.01	-
	**a_w_ Ø**					
**Salami**	0.96 ± 0.00	-	0.95 ± 0.00	0.94 ± 0.00	-	0.95 ± 0.00
**Mettwurst**	0.97 ± 0.00	0.96 ± 0.00	-	0.96 ± 0.00	0.95 ± 0.00	-

## Data Availability

The original contributions presented in the study are included in the article/Supplementary Material, further inquiries can be directed to the corresponding author.

## Supplementary Materials

The following supporting information can be downloaded at: https://www.mdpi.com/article/10.3390/foods13020298/s1, Table S1. List of bacteriocins and the primers used for the screening of their structural genes.
