# Supplementary material for: Growth Control of Listeria monocytogenes in Raw Sausage via Bacteriocin-Producing Leuconostoc carnosum DH25
Source: Foods. 2024 Jan 17;13(2):298. doi: 10.3390/foods13020298 (PMC10815048; doi:10.3390/foods13020298)
Supplement: Supplementary file 1 [file foods-13-00298-s001.zip › foods-2814103-supplementary.pdf]

Supplementary Material

Table S1. List of bacteriocins and the primers used for the screening of their structural genes.

Target bacteriocin	Primers	Sequence (5′→3′)	AT (°C)	Bibliography
Plantaricin S	PlanS – F<br>PlanS - R	GCCTTACCAGCGTAATGCCC<br>CTGGTGATGCAATCGTTAGTTT	50	Stephens et al., 1998
Plantaricin NC8	PlanNC8 - F<br>PlanNC8 - R	GGTCTGCGTATAAGCATCGC<br>AAATTGAACATATGGGTGCTTTAAATTCC	55	Maldonado et al., 2003
Plantaricin W	PlanW – F<br>PlanW – R	TCACACGAAATATTCCA<br>GGCAAGCGTAAGAAATAAATGAG	55	Holo et al., 2001
Sakacin G	skgA2 – F<br>skgA2 - R	CGTTACAACAGAACTTCAAG<br>TGGAAGAATGAGTACTTGTT	50	Todorov et al., 2011
Plantaricin 1.25β	plnB – F<br>plnB – R	TAGCATTGATTGATGGAGGAAA<br>GCATGCCGTGTAAGTTGTTAGA	55	Ehrmann et al., 2000<br>Macwana and Muriana, 2012
Sakacin X	sakX – F<br>sakX – R	AGCTATGAAAGGTATTGTCGGG<br>TAAGATTTCCAGCCAGCAGC	53	Macwana and Muriana, 2012
Sakacin T	Sak T α – F<br>Sak T α – R<br>Sak T β – F<br>Sak T β - R	TCGGTGGCTATACTGCTAAACA<br>TGTCCTAAAAATCCACCAATGC<br>AAGAAATGATAGAAATTTTTGGAGG<br>TGTGAAATCCAATCTTGTCCTG	53	Macwana and Muriana, 2012
Pediocin PA-1/AcH	Ped – F<br>Ped – R	GGTAAGGCTACCACTTGCAT<br>CTACTAACGCTTGGCTGGCA	55	Suwanjinda et al., 2007
Plantaricin EF	Pln – F<br>Pln – R	GGCATAGTTAAAATTCCCCCC<br>CAGGTTGCCGCAAAAAAAG	55	Rojo-Bezares et al., 2007<br>Rizzello et al., 2014
Curvacin A	CurA – F<br>CurA - R	GTAAAAGAATTAAGTATGACA<br>TTACATTCCAGCTAAACCACT	50	Remiger et al., 1996
Sakacin P	Sak P – F<br>Sak P - R	ATGGAAAAGTTTATTGAATTA<br>TTATTTATTCCAGCCAGCGTT	50	Remiger et al., 1996
Plantaricin A	Pln A – F<br>Pln A - R	GTACAGTACTAATGGGAG<br>CTTACGCCATCTATACG	53	Remiger et al., 1996
Sakacin Q	Sak Q – F<br>Sak Q – R	ATGCAAAATACAAAAGAACTAA<br>CGCTTGTTTAGAGACACCCGTT	50	Cocolin and Rantsiou, 2007
Plantaricin JK	Pln J – F<br>Pln J – R	TAACGACGGATTGCTCTG<br>AATCAAGGAATTATCACATTAGTC	51	Rojo-Bezares *et al.*, 2007
	Pln K – F<br>Pln K - R	AATCGCAGTGACTTCCAGAAC<br>AGAGCAATCCGTCGTTAATAAATG	53.7	Anderssen *et al.*, 1998
Nisin	For<br>Rev	AAGAATCTCTCATGAGT<br>CCATGTCTGAACTAACA	41	Garde *et al.*, 2000
